# Umbilical Cord Hernia and Meckel's Diverticulum: Beware of the Umbilical Clamping!

**DOI:** 10.1055/a-2747-7295

**Published:** 2025-12-06

**Authors:** Taisia Bollettini, Thibault Planchamp, Solène Joseph, Luana Carfagna, Olivier Abbo

**Affiliations:** 1 Department of Medical, Surgical and Neurological Sciences, University of Siena, Siena, Italy; 2 Service de Chirurgie - Hopital des Enfants, CHU Toulouse, Toulouse, France

**Keywords:** umbilical cord hernia, Meckel's diverticulum, umbilical clamping, neonatal occlusion

## Abstract

Congenital hernia in the umbilical cord is a rare form of ventral abdominal wall defect, which, if not diagnosed, can lead to iatrogenic intestinal injuries due to improper umbilical clamping. We report a case of a newborn referred to our center for intestinal obstruction caused by a decapitated Meckel's diverticulum located within the umbilical cord. Only a few similar cases have been documented in the literature, including four fatalities. Our aim is to present this rare case to the surgical community to raise awareness about it as a potential differential diagnosis in neonatal obstruction cases and emphasize the importance of early treatment to reduce the risk of high morbidity and even mortality.

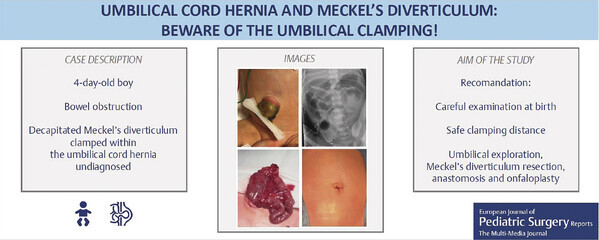

## Introduction

Umbilical cord hernia (UCH) is a rare condition in pediatric surgery, occurring in approximately 1 in 5,000 live births. 1 This uncommon congenital abdominal wall defect, usually less than 4 cm in size, typically contains only the midgut and is covered by a membrane (Rathke's membrane continuous with the parietal peritoneum), Wharton's jelly, and a thin amniotic layer. 1 It results from the failure of the midgut to return to the peritoneal cavity between 10 and 12 weeks of gestation. A careful evaluation of the umbilical cord at birth can facilitate the diagnosis of even the smallest defects, allowing for prompt and effective treatment. The bowel can often be easily reduced into the peritoneal cavity, the fascia can be closed, and cosmetic umbilicoplasty is almost always feasible.

Conversely, if this congenital anomaly is not identified during the clinical examination at birth, improper cord clamping may occur, potentially leading to intestinal damage. We report a case of accidental umbilical clamp decapitation of a Meckel's diverticulum in a small UCH.

Due to the extreme rarity of this case report, it is not possible to propose effective and safe diagnostic and therapeutic management strategies based solely on our experience. Therefore, we conducted a thorough review of the literature to better understand and identify the most appropriate approaches. The review revealed only a few similar cases, including four fatalities related to the delayed diagnosis of intestinal perforations. Diagnosis of iatrogenic intestinal injury in these cases can be challenging and insidious, with rapid deterioration of the patient's clinical condition. By comparing our findings with previously reported cases, we aimed to provide a more comprehensive perspective on optimal management strategies.

## Case Description

After an uncomplicated pregnancy, a 4-day-old boy, born at 38 weeks of gestation with a birth weight of 2,660 g, was referred to our center for bowel obstruction. Meconium was passed within the first 24 hours. On the second day of life, he developed bilious emesis, abdominal distension, and constipation.

**Fig. 1:**
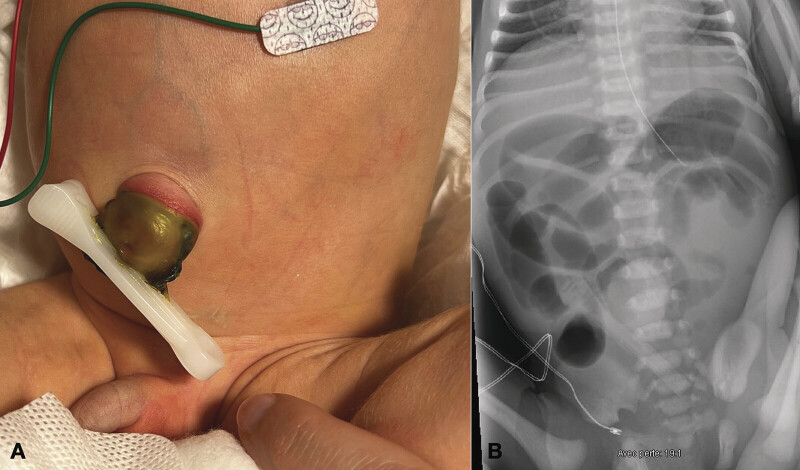
( **A** ) Inflamed umbilical cord, clamped approximately 2.5 cm from the umbilical base. ( **B** ) Abdominal X-ray showing a small bowel obstruction without pneumoperitoneum.

Clinical examination revealed an inflamed umbilical cord, clamped approximately 2.5 cm from the umbilical base, with crepitus on palpation and bowel sounds on auscultation ( Fig. 1A ). The abdomen was distended. An abdominal X-ray revealed a small bowel obstruction without pneumoperitoneum ( Fig. 1B ), and an ultrasound identified a UCH with a small bowel segment clamped by the umbilical clamp.

**Fig. 2:**
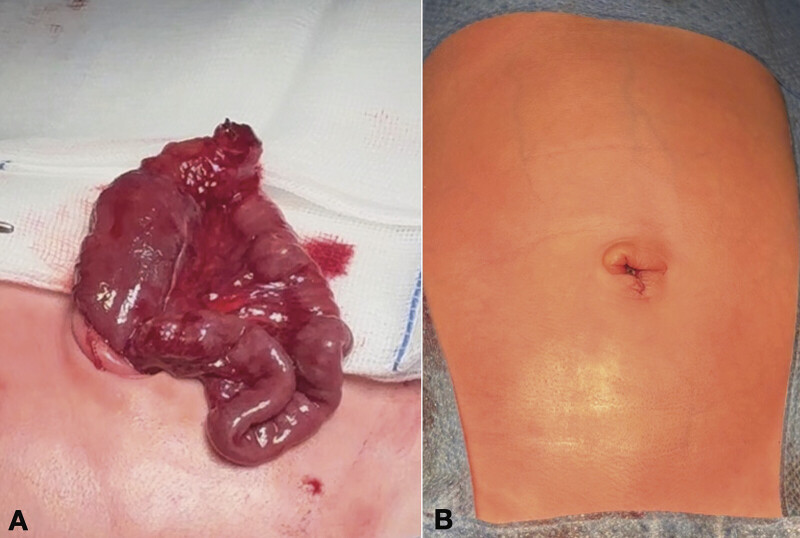
( **A** ) Umbilical exploration revealed a perforated Meckel's diverticulum. ( **B** ) Final aspect of omphaloplasty.

Under general anesthesia, umbilical exploration was performed with a semicircular incision around the base of the umbilical cord, avoiding laparotomy. This revealed a clamped and perforated Meckel's diverticulum ( Fig. 2A ). The diverticulum was resected, and an end-to-end intestinal anastomosis was performed. The abdominal wall defect, approximately 1.5 cm in size, was closed, followed by an omphaloplasty ( Fig. 2B ).

The histopathological analysis of the resected intestine (2 cm in length) confirmed the diagnosis of Meckel's diverticulum with intestinal mucosa without ectopic tissue.

After a 48-hour course of antibiotics (C3G) and a 5-day fasting period, feeding was gradually reintroduced, and the patient was discharged on postoperative day 15. No complications were observed after 6 months of follow-up.

## Discussion

The insidious association between UCH and the presence of a Meckel's diverticulum was first described in the literature in 1996 by Jona, 2 who reported six cases over a period of 10 years. He emphasized the importance of paying attention to any enlargements or swellings of the umbilical cord and ensuring that it is clamped at a safe distance, with early pediatric surgical consultation recommended. He explained that when UCH is associated with a patent omphalomesenteric duct, the ileum is anchored in an extra-abdominal position within the hernia and is at risk of being caught in the umbilical clamp.

Three similar cases were described in the literature afterward. In 2006, van Tuil et al 3 reported a case of accidental decapitation of the omphalomesenteric duct by clamping an undiagnosed UCH approximately 7 cm from the umbilical base after delivery. This is the longest distance of clamp described in the literature related to this iatrogenic injury. In the case reported by Uri et al 4 in 2008, the cord was clamped approximately 3 cm from the umbilical base, and in the most recent case described by Zvizdic et al 5 in 2021, the cord was clamped approximately 2.5 cm from the base of the umbilical cord, similar to our own case. Due to the rarity of this condition, it is not possible to scientifically define a precise safe distance for clamping.

Certainly, to prevent this type of intestinal damage, it is essential to educate health care staff involved in recognizing this rare pathological entity. This training will enable them to carefully examine the umbilical cord and, if suspicious, request a specialist consultation, clamp at a safe distance, perform an ultrasound to confirm the diagnosis, and provide appropriate treatment.

If this iatrogenic intestinal damage occurs, it can manifest as immediate meconium leakage from the clamped umbilical cord or as progressively developing intestinal obstruction, which can be extremely dangerous for the infant.

In a 2008 review by Asabe et al 6 on the accidental clamping or cutting of a hernia in the umbilical cord, a total of 16 patients were included, 2 of whom had a Meckel's diverticulum. Four of these patients died due to severe complications caused by intestinal obstruction.

When the clinical presentation is neonatal occlusion without meconium in the umbilical stump, the condition can be confirmed with ultrasound or a lateral abdominal radiograph showing intestinal air in the umbilical cord. This enables surgical intervention to explore the umbilical cord, potentially avoiding a laparotomy.

All 10 reported cases in the literature ( Table 1 ) where iatrogenic damage from umbilical hernia clamping was associated with a Meckel's diverticulum were treated with umbilical cord exploration. Five patients (50%) were treated with a Meckel's excision and transverse ileal suture, and the remaining cases, including ours, were treated with intestinal resection and end-to-end anastomosis. No complications were reported in any of these cases, and the aesthetic outcomes were extremely satisfactory.

**Table 1:** Characteristics of the only 10 cases of accidental Meckel's decapitation by clamping an undiagnosed umbilical cord hernia reported in the literature to date

Study	Number of patients	Median age (days)	Sex	Distance of the clamp	Clinic	Imaging	Treatment	Complications
Bollettini et al (this study), 2025	1	4	M	2.5 cm	Bowel obstruction	US RX	IR end-to-end anastomosis	None
Zvizdic et al, 2021 5	1	2	M	2.5 cm	Bowel obstruction	RX	ME transverse suture	None
Uri et al, 2008 4	1	0	M	3 cm	Meconium in the umbilical stump	–	ME transverse suture	None
van Tuil et al, 2006 3	1	0	M	7 cm	Bowel loop in UCH	–	ME transverse suture	None
Jona, 1996 2	6	2.8	M	1 pt: 1.5 cm 4 pt <1 cm 1 pt: 3 cm	5 pt: Meconium in the umbilical stump 1 pt: Bowel loop in UCH	–	4 pt: IR end-to-end anastomosis 2 pt: Meckel excision and transverse suture	None

Abbreviations: IR, intestinal resection; ME, Meckel excision; Pt, patient; RX, radiography; UCH, umbilical cord hernia; US, ultrasound.

## Conclusions

Due to its rarity and lack of awareness, UCH can be easily misdiagnosed, leading to inappropriate umbilical clamping with the risk of intestinal perforation. UCH with a herniated and clamped Meckel's diverticulum is even rarer, and only a few cases have been described in the literature.

This report aims to raise awareness and provide essential information to help clinicians suspect congenital umbilical hernia and prevent iatrogenic bowel injury. Since improper clamping can have fatal consequences for a newborn, it is crucial to thoroughly examine the umbilical cord at birth, paying close attention to any swelling or unusual shapes. In such suspicious cases, maintaining a safe distance when clamping and seeking a prompt pediatric surgical evaluation are key steps. If an injury is suspected, a minimal umbilical exploration may be considered a potential alternative to a full laparotomy, as it could offer safe outcomes while preserving excellent cosmetic results, although further evidence is needed to confirm these benefits.

## Notes

Importance for a Pediatric Surgeon Early recognition of umbilical cord hernia complications, such as Meckel's diverticulum injury, allows prompt surgical intervention, reducing morbidity and mortality while ensuring safe and functional outcomes.
